# Relationship between hemoglobin A1c and cardiovascular disease in mild-to-moderate hypercholesterolemic Japanese individuals: subanalysis of a large-scale randomized controlled trial

**DOI:** 10.1186/1475-2840-10-58

**Published:** 2011-06-30

**Authors:** Rimei Nishimura, Tomoko Nakagami, Hirohito Sone, Yasuo Ohashi, Naoko Tajima

**Affiliations:** 1 Division of Diabetes, Metabolism and Endocrinology, Department of Internal Medicine, Jikei University School of Medicine, 3-25-8 Nishi-Shinbashi, Minato-ku, Tokyo, 105-8461, Japan; 2 Diabetes Center, Tokyo Women's Medical University, 8-1 Kawada-cho, Shinjuku-ku, Tokyo, 162-8666, Japan; 3 Department of Internal Medicine, University of Tsukuba Institute of Clinical Medicine, 3-2-7 Miya-machi, Mito, Ibaraki, 310-0015, Japan; 4 Department of Biostatistics/Epidemiology and Preventive Health Sciences, University of Tokyo, 7-3-1 Hongo, Bunkyo-ku, Tokyo, 113-8654, Japan; 5 Jikei University School of Medicine, 3-25-8 Nishi-Shinbashi, Minato-ku, Tokyo, 105-8461, Japan

**Keywords:** Hemoglobin A1c (HbA1c), cardiovascular disease (CVD), hypercholesterolemia, HMG CoA reductase inhibitor, pravastatin, MEGA Study

## Abstract

**Background:**

Although the ADA/EASD/IDF International Expert Committee recommends using hemoglobin A1c (HbA1c) to define diabetes, the relation between HbA1c and cardiovascular disease (CVD) has not been thoroughly investigated. We analyzed this relation using clinical data on Japanese individuals with hypercholesterolemia.

**Methods:**

In the large-scale MEGA Study 7832 patients aged 40 to 70 years old with mild-to-moderate hypercholesterolemia without CVD were randomized to diet alone or diet plus pravastatin and followed for >5 years. In the present subanalysis of that study a total of 4002 patients with baseline and follow-up HbA1c data were stratified according to having an average HbA1c during the first year of follow-up <6.0%, 6.0%-<6.5%, or ≥6.5% and their subsequent 5-year incidence rates of CVD compared according to sex, low-density lipoprotein cholesterol (LDL-C), and treatment arm.

**Results:**

Overall, risk of CVD was significantly 2.4 times higher in individuals with HbA1c ≥6.5% versus <6.0%. A similar relation was noted in men and women (hazard ratio [HR], 2.1; p <0.01 and HR, 3.0; p <0.01, respectively) and was regardless of treatment arm (diet alone group: HR, 2.2; p <0.001; diet plus pravastatin group: HR, 1.8; p = 0.02). Spline curves showed a continuous risk increase according to HbA1c level in all subpopulations studied.

**Conclusions:**

In hypercholesterolemic individuals the risk of CVD increases linearly with HbA1c level. This significant contribution by elevated HbA1c to increased CVD is independent of pravastatin therapy, and thus requires appropriate HbA1c management in addition to lipids reduction.

## Background

Clinical markers such as fasting plasma glucose (FPG), oral glucose tolerance test, and postprandial glucose are commonly used to define diabetes or impaired glucose tolerance [1-3]. However, in 2009 an International Expert Committee assembled by the American Diabetes Association (ADA), European Association for the Study of Diabetes (EASD), and International Diabetes Federation (IDF) announced that HbA1c assay is a more reliable marker to diagnose diabetes, using a cutpoint of ≥6.5% [4]. Observational studies indicate that measuring HbA1c, compared with a single measure of glucose concentration, better captures the impact of long-term glycemic exposure and severity of diabetic complications. Epidemiologic data have also demonstrated relations between HbA1c and microvascular and macrovascular disease [5-7]. On the other hand, data are scarce for patients with a specific disease background such as hypercholesterolemia.

This subanalysis evaluated the relation between HbA1c and CVD using data from the long-term, large-scale Management of Elevated Cholesterol in the Primary Prevention Group of Adult (MEGA) Study conducted in Japan in the late 1990s to mid-2000s [8,9].

## Methods

The design, baseline characteristics, and major outcomes of the prospective, randomized, open-label, blinded endpoints MEGA Study have been described previously [8,9]. Briefly, 7832 patients (2476 men and 5356 postmenopausal women) aged 40-70 years with mild-to-moderate hypercholesterolemia (total cholesterol [TC] level, 5.7-6.0 mmol/L) without CVD were randomly allocated to either the diet group or diet plus pravastatin (10-20 mg/day, the approved dose in Japan) group for a mean follow-up period of 5.3 years. Patients in both arms were counseled to follow the National Cholesterol Education Program (NCEP) Step I diet [10] throughout the study period. Pravastatin was initiated at 10 mg/day; however, if the TC level did not decrease to ≤5.7 mmol/L the dosage could be uptitrated to 20 mg/day, in compliance with Japanese dosing instructions. Patients whose TC remained >5.7 mmol/L, even after enhancement of assigned treatment, could be switched to other aggressive treatments including other statins. Concomitant treatment for complications was not restricted in both groups. The primary composite endpoint was first occurrence of coronary heart disease (CHD) comprising fatal and nonfatal myocardial infarction, angina pectoris, cardiac/sudden death, and coronary revascularization procedure. Secondary endpoints included all strokes, CHD plus ischemic stroke, all CVD events, and total mortality.

Patients were evaluated by their attending physician at 1, 3, and 6 months and every 6 months thereafter. Health checkups at each clinic visit included biochemical tests, including HbA1c levels, and assessment of patient adherence to treatment. For each event, detailed information was obtained from physicians and evaluated by the blinded Endpoints Committee according to established criteria. TC, high-density lipoprotein cholesterol (HDL-C), triacylglycerides (TG), and lipoprotein(a) (Lp[a]) levels were centrally measured at the same laboratory using methods standardized by the Centers for Disease Control and Prevention (Atlanta, GA). Low-density lipoprotein cholesterol (LDL-C) level was estimated by Friedewald's formula. Fasting glucose, HbA1c, and other laboratory values were measured at each participating institution. Because the Japan Diabetes Society (JDS) method was used to measure HbA1c, which has values that are 0.3%-0.4% percent lower than National Glycohemoglobin Standardization Program (NGSP) values [11], for this report we converted all HbA1c values to NGSP values by the following formula: HbA1c (%) = 0.0981 × International Federation of Clinical Chemistry (IFCC) value (mmol/L) [10.39 × JDS value (%) - 16.8] + 1.95 [12].

We analyzed data on patients whose HbA1c value was determined during the first 12 months of the study (5 values per patient; baseline, and at 1, 3, 6 and 12 months). Using these data, we assessed relations between average HbA1c value for the first 12 months and occurrence of CVD events including myocardial infarction, angina, cardiac and sudden death, a coronary revascularization procedure, stroke, transient ischemic attack, and arteriosclerosis obliterans occurring during the 5 years of follow-up. Event rates were compared for three categories of HbA1c levels such as <6.0%, 6.0%-<6.5%, and ≥6.5% using the new criteria from the International Expert Committee [4]. Hazard ratios (HRs) and 95% confidence intervals (95%CIs) were estimated by multivariate Cox proportional hazards model. Relations between HbA1c and risk of CVD events were evaluated by multivariable Cox proportional hazards model with restricted quadratic spline [13]. The three knots for quartiles were adopted as optimal analysis model for this study, based on the results of comparison of a model using two and four knots for HbA1c tertiles and quintiles and that using three knots for 5.0%, 6.0%, and 7.0% of HbA1c. The multivariate models were simultaneously adjusted by sex, age, treatment arm, baseline LDL-C, baseline HDL-C, hypertension, chronic kidney disease (estimated glomerular filtration rate <60), and smoking status.

## Results

Baseline clinical and demographics characteristics of the 4002 patients included in this analysis are summarized in Table 1. Two thirds of the patients (66%) were women; men were about 4 years younger than women and had a slightly higher BMI. Confirmed diabetes, and consequently average FPG and HbA1c, were higher in men, whereas confirmed hypertension was higher in women. LDL-C and HDL-C were somewhat lower, and TG higher, in men than women.

**Table 1:** Baseline characteristics of patients

Variable	Men	Women	Total
*n*	1374	2628	4002
Age, years	55.5 ± 8.1	59.8 ± 6.0	58.3 ± 7.1
BMI, kg/m^2^	24.1 ± 2.8	23.8 ± 3.4	23.9 ± 3.2
Diabetes, *n *(%)	626 (45.6)	870 (33.1)	1496 (37.4)
Hypertension, *n *(%)	514 (37.4)	1108 (42.2)	1622 (40.5)
Current smoker, *n *(%)	518 (37.7)	145 (5.5)	663 (16.6)
TC, mmol/L	6.24 ± 0.31	6.29 ± 0.32	6.27 ± 0.32
LDL-C, mmol/L	4.01 ± 0.47	4.08 ± 0.43	4.06 ± 0.45
HDL-C, mmol/L	1.36 ± 0.36	1.54 ± 0.38	1.48 ± 0.38
Non-HDL, mmol/L	4.88 ± 0.46	4.76 ± 0.48	4.80 ± 0.48
TG (median [IQR]), mmol/L	1.71 (1.26-2.45)	1.33 (1.01-1.80)	1.44 (1.08-2.01)
FPG, mmol/L	6.80 ± 2.27	6.15 ± 1.80	6.37 ± 2.00
HbA1c, %	6.47 ± 1.38	6.22 ± 1.16	6.31 ± 1.24
Oral hypoglycemic agent, *n *(%)	291 (21.2)	423 (16.1)	714 (17.8)
Insulin, *n *(%)	55 (4.0)	98 (3.7)	153 (3.8)

Values are mean ± SD unless otherwise indicated.

Diabetes and hypertension defined by physician diagnosis. BMI, body mass index; TC, total cholesterol; LDL-C, low-density lipoprotein cholesterol; HDL-C, high-density lipoprotein cholesterol; TG, triacylglycerides; FPG, fasting plasma glucose; IQR, interquartile range.

The incidence of cardiovascular events in men and women was compared among the three categories of HbA1c levels as shown in Table 2. Comparing the ≥6.5% versus <6.0% HbA1c group overall, CHD was significantly higher (HR, 3.1; p <0.01), and also in men (HR, 2.3; p <0.01) and women (HR, 4.9; p <0.01). Moreover, CVD was significantly increased (HR, 2.4; p <0.01), and in men (HR, 2.1; p <0.01) and women (HR, 2.9; p <0.01). As the HbA1c level increased, the incidence of CHD and CVD increased. Whereas, stroke incidence was slightly higher in the HbA1c ≥6.5% group for the total population and in men and in women, although it did not reach statistical significance. In men, but not women, there was a significant trend for increased mortality as HbA1c levels increased.

**Table 2:** Incidence of CVD events in relation to HbA1c level

% HbA1c	Men				Women				All			
	**Events/pts, n (/1000 py)**	**HR (95%CI)**	**p-value**	**Trend-p**	**Events/pts, n (/1000 py)**	**HR (95%CI)**	**p-value**	**Trend-p**	**Events/pts, n (/1000 py)**	**HR (95%CI)**	**p-value**	**Trend-p**

CHD				<0.01				<0.01				<0.01
<6.0	16/630 (5.6)	1.0	--		9/1481 (1.3)	1.0	--		25/2111 (2.6)	1.0	--	
6.0-< 6.5	7/226 (7.1)	1.2 (0.5-2.9)	0.73		6/419 (3.2)	2.1 (0.7-6.0)	0.16		13/645 (4.5)	1.5 (0.8-2.9)	0.24	
≥6.5	33/518 (14.7)	2.3 (1.3-4.2)	<0.01		24/728 (7.2)	4.9 (2.2-10.6)	<0.01		57/1246 (10.3)	3.1 (1.9-5.0)	<0.01	
Stroke (all)				0.33				0.14				0.07
<6.0	11/630 (3.9)	1.0	--		13/1481 (1.9)	1.0	--		24/2111 (2.5)	1.0	--	
6.0-< 6.5	5/226 (5.0)	1.1 (0.4-3.3)	0.82		4/419 (2.1)	1.0 (0.3-3.0)	0.94		9/645 (3.1)	1.1 (0.5-2.3)	0.85	
≥6.5	15/518 (6.5)	1.5 (0.7-3.2)	0.33		14/728 (4.2)	1.8 (0.8-3.9)	0.13		29/1246 (5.1)	1.7 (1.0-2.9)	0.07	
CVD				<0.01				<0.01				<0.01
<6.0	27/630 (9.6)	1.0	--		26/1481 (3.9)	1.0	--		53/2111 (5.6)	1.0	--	
6.0-< 6.5	12/226 (12.2)	1.2 (0.6-2.3)	0.69		10/419 (5.4)	1.2 (0.6-2.5)	0.64		22/645 (7.7)	1.2 (0.7-2.0)	0.48	
≥6.5	50/518 (22.9)	2.1 (1.3-3.4)	<0.01		42/728 (12.8)	2.9 (1.8-4.7)	<0.01		92/1246 (16.9)	2.4 (1.7-3.4)	<0.01	
Death (total)				0.01				0.53				0.04
<6.0	4/630 (1.4)	1.0	--		16/1481 (2.3)	1.0	--		20/2111 (2.1)	1.0	--	
6.0-< 6.5	5/226 (4.9)	3.4 (0.9-12.9)	0.07		10/419 (5.2)	2.2 (1.0-4.9)	0.05		15/645 (5.1)	2.3 (1.2-4.4)	0.02	
≥6.5	16/518 (6.7)	4.1 (1.3-12.5)	0.01		10/728 (2.9)	1.2 (0.5-2.6)	0.68		26/1246 (4.5)	1.9 (1.0-3.4)	0.04	

HR, 95%CI estimated by Cox proportional model adjusted by sex, age, treatment arm, baseline LDL-C, baseline HDL-C, hypertension, chronic kidney disease (estimated glomerular filtration rate <60), and smoking status.

**Figure 1:**
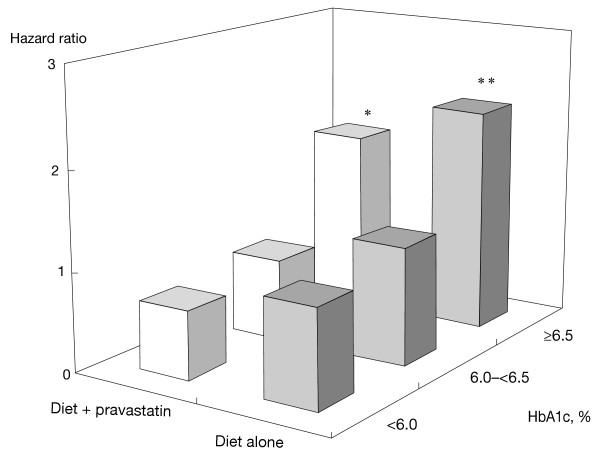
**Relation between HbA1c and CVD according to treatment arms**. P-values were calculated against <6.0% in each group by Cox proportional hazard model adjusted for sex, age, baseline LDL-C, baseline HDL-C, hypertension, chronic kidney disease (estimated glomerular filtration rate <60), and smoking. *p <0.05; **p <0.01.

**Figure 2:**
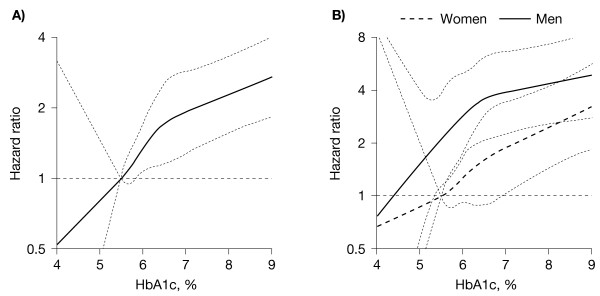
**Spline curves for CVD in total population (A) and in men and women (B).** Knots are quartiles of HbA1c in the total population. A) Dashed lines = 95%CI. B) Solid lines = 95%CI for men; dashed lines = women.

Comparing patients whose HbA1c was ≥6.5% versus <6.0% by treatment arm revealed that CVD was significantly increased in the diet alone group (HR, 2.2, p <0.001) and similarly in diet plus pravastatin group (HR, 1.8; p *= *0.02; Figure 1) in patients with HbA1c ≥6.5%. The spline curves for CVD exhibited a linear increase in risk as HbA1c increased until approximately HbA1c <6.0%; thereafter the risk increased more gently (Figure 2). Although CVD risk was proportionally lower in women than men, no large difference in the shape of the spline curves was found between the two sexes.

## Discussion

We found a significant relation between having an HbA1c level ≥6.5% and risk of CVD in Japanese patients with hypercholesterolemia. This analysis compared three categories of HbA1c levels as measured according to new International Expert Committee criteria. Significant relations between increased HbA1c level and CHD and CVD were observed in men and women. A continuous increase in CVD risk as HbA1c level rose was confirmed by the spline method, which indicated that there is no threshold between CVD risk and HbA1c level. Recent reports have demonstrated that including HbA1c level in the assessment model could help to identify subjects at high risk, and that the predictability was improved by also including lipid levels [14]. Therefore, evaluating the relationship between HbA1c and CVD in patients with hypercholesterolemia is important to identify patients in a high-risk population.

The incidence of microvascular disease is known to increase with higher HbA1c levels. For instance, diabetic retinopathy occurs at a high rate in persons with HbA1c >6.0%-7.0% [15]. The Kumamoto Study in Japan demonstrated that the risk of retinopathy and nephropathy starts to increase as HbA1c rises above 6.6%-6.7% (NGSP) [16]. Macrovascular disease also is affected by a high HbA1c. In the Framingham Heart Study, a 1% increase in HbA1c was associated with a 1.39-fold increased risk of CVD [17]. A graded association between HbA1c and carotid intima-media thickness was found in the Atherosclerosis Risk in Communities (ARIC) study [18]. The Suita Study [11] recently reported a significantly increased risk of CVD associated with high HbA1c. Although it appears clear from these epidemiologic data and interventional studies that increased HbA1c levels are associated with raised CVD risk, we believe that the present findings are the first such demonstration in the setting of hypercholesterolemia, which itself confers high risk for CVD. Our data indicate that the significant relation between CVD risk and HbA1c ≥6.5% observed in hypercholesterolemia is similar to that seen in the general population.

In our previous report, we stated that the incidence of CVD in patients with or without abnormal fasting glucose was 16.5 and 5.7 per 1000 patient-years, respectively. In the present study, the incidences of CVD in patients with HbA1c ≥ 6.1% and <6.1% were 16.9 and 6.1 per 1000 patient-years, respectively [19]. Although the results are broadly consistent when using fasting glucose and HbA1c as markers for glucose abnormalities to determine the HRs, 5% of the patients with HbA1c ≥6.1% actually had normal fasting glucose levels (data not shown). Since the HbA1c level is affected by postprandial glucose excursions more than the fasting glucose level [20], evaluating HbA1c in addition to fasting glucose is useful to identify people at high risk.

Statins can reduce CVD onset in the settings of primary and secondary prevention [21-25]. Benefits of statin therapy have been reported in patients with diabetes [9,19,26] and confirmed in a large-scale meta-analysis that implied that statins reduce CVD risk in diabetic and nondiabetic populations [27]. In this study, although statistical significance was not reached probably due to the small number of events, we nonetheless observed a proportional risk reduction in each HbA1c category in the diet plus pravastatin group compared with diet alone group (in patients with HbA1c <6.0%, 6.0%-<6.5%, and ≥6.5%, HR = 0.64, 0.72, and 0.81, respectively). National guidelines recommend the aggressive management of CVD in patients with type 2 diabetes and that multidisciplinary interventions that includes weight, lipid and blood pressure control are needed to minimize the CVD risk, in addition to glucose lowering[28]. Statins offer an important therapeutic option for the management of lipids, particularly in type 2 diabetes. However, the relation between HbA1c and CVD was unaltered suggesting that appropriate HbA1c management is required in addition to lipids reduction. There are some hypotheses for the mechanisms by which hydrophilic statins may attenuate the deterioration of abnormal glucose metabolism in diabetic patients [29]. However, the HbA1c level was not significantly different between the diet plus pravastatin group and the diet alone group throughout the study period, as we have previously reported [19]. It is possible that adherence to diet therapy was somewhat worse in the diet plus pravastatin group than in the diet alone group, which may or may not influence the HbA1c level. However, we do not think we should focus on the effects of pravastatin on HbA1c levels in this analysis because there was no difference in HbA1c levels between the two groups.

There are two important limitations to the present analysis. First, it was conducted in patients whose TC was 5.7-6.0 mmol/L, and extrapolating these results to patients with higher TC concentrations should be done with caution. On the other hand, our patients had a broad range of LDL-C levels (1.4-4.6 mmol/L). Thus it appears that our findings may be quite generalizable. Second, the small number of events in the MEGA Study may affect the accuracy of the spline method. Therefore the tail analysis of data for sex, LDL-C level, and treatment arm were exploratory, especially in sparse tail area. In addition, an earlier study showed that all-cause mortality was increased in people with low HbA1c levels (<4.0%) [30]. Although we cannot assess the relationship between low HbA1c and high mortality because few patients with low HbA1c levels were included in this analysis, we should take this into account in clinical practice.

In conclusion, our results suggest that the HbA1c level should be included in prediction models for CVD risk. Controlling HbA1c independently of lipid management is necessary to reduce the risk of CVD in diabetic patients with elevated HbA1c.

## Competing interests

The authors declare that they have no competing interests.

## Authors' contributions

RN carried out interpreting the data and writing the manuscript; TN, HS and NT carried out interpreting the data and reviewing the manuscript; YO carried out analyzing and reviewing the manuscript. All authors read and approved the final manuscript.
